# Cortical Laminar Necrosis as a Rare Complication of Streptococcus pneumoniae Meningitis: A Case Report

**DOI:** 10.7759/cureus.68086

**Published:** 2024-08-29

**Authors:** Subbiah Senthilnathan, Gunasekaran Nallusamy, Priyadarshini Varadaraj, Keesari Sai Sandeep Reddy, Chakradhar Ravipati

**Affiliations:** 1 Internal Medicine, Saveetha Medical College and Hospital, Saveetha Institute of Medical and Technical Sciences (SIMATS) Saveetha University, Chennai, IND; 2 Radiodiagnosis, Saveetha Medical College and Hospital, Saveetha Institute of Medical and Technical Sciences (SIMATS) Saveetha University, Chennai, IND

**Keywords:** cerebral infarct, streptococcus pneumoniae, neurological deficits, bacterial meningitis, cortical laminar necrosis

## Abstract

Cortical laminar necrosis (CLN) is a rare neurological complication that refers to ischemic injury of selective neuronal cortical layers. This condition often gets triggered by hypoxia, hypoglycemia, status epilepticus, immunosuppressive therapy, and rarely infection. This case report highlights the clinical presentation, diagnostic challenges, management, and outcomes of a patient who developed CLN due to bacterial meningitis. A 54-year-old woman with no significant medical history presented with high-grade fever, vomiting, and headache for two days. The clinical findings and cerebrospinal fluid (CSF) analysis indicated bacterial meningitis, leading to the initiation of empirical intravenous antibiotics. Despite initial improvement with antibiotics, the patient's condition worsened on day four, and she presented with increased headache and dizziness. An MRI performed on day four revealed CLN. *Streptococcus pneumoniae* was subsequently identified as the causative agent, and the antibiotic regimen was escalated based on the CSF culture and sensitivity results. By day nine, the patient experienced pain relief and a fever reduction. Although there were initial cognitive deficits, these improved significantly by the end of the second week with conservative management. The patient was discharged at the end of the second week, with a follow-up brain MRI scheduled one month later. This case highlights the critical importance of early recognition and aggressive management of bacterial meningitis to prevent neurological complications such as CLN. MRI plays a key role in neuroprotection for patients with CLN. Long-term follow-up and optimal antibiotic therapy are essential for safeguarding patient outcomes and ensuring quality of life.

## Introduction

Bacterial meningitis is a potentially life-threatening and acute inflammation of the protective membranes covering the brain and spinal cord. It can lead to severe complications such as cerebral edema, elevated intracranial pressure, seizures, and chronic neurological damage. The most common pathogens causing bacterial meningitis are *Streptococcus pneumoniae*, *Neisseria meningitidis*, and *Haemophilus influenzae* [1]. The pathogenesis is multifactorial, involving factors such as pathogen invasion, toxin-induced inflammation, and hypoxic-ischemic injuries in neuronal regions [2]. While bacterial meningitis primarily presents with symptoms such as fever, headache, and neck stiffness, its progression can result in substantial neurological damage if not promptly and effectively treated. It can have far-reaching consequences beyond its immediate symptoms, with one severe complication being cortical laminar necrosis (CLN). CLN is a rare but devastating complication of bacterial meningitis that affects the brain's cortical structures, leading to severe neurological implications. It is characterized by the selective degeneration of neurons within specific layers of the cerebral cortex [3]. Histologically, gray matter is more susceptible to necrosis from oxygen deprivation than white matter. Within the gray matter, the third layer is the most vulnerable. Additionally, damage tends to be more pronounced in the sulci compared to the gyri of the brain. It may lead to the death of neurons, glial cells, and associated blood vessels at the cortical laminae. These lesions can cause persistent neurological deficits even if the triggering etiology is treated.

## Case presentation

A 54-year-old woman with no significant medical history presented to the emergency department with a two-day history of high-grade fever, altered mental status, and headache. The headache was diffuse, moderate to severe, and did not improve with analgesics. She also experienced four episodes of vomiting. However, she had no visual disturbances, seizures, or focal neurological symptoms. Additionally, she did not have other symptoms such as ear discharge, hearing loss, shortness of breath, cough with sputum, painful urination, diarrhea, or bleeding tendencies. There was no history of similar episodes previously.

Clinical findings and microbiological confirmation

The patient was awake but drowsy, disoriented to time, place, and person, and only responded to painful stimuli. Despite stable vital signs, her Glasgow Coma Scale (GCS) score was notably low at E1V1M4. Her blood glucose level upon admission was 167 mg/dL. Due to the low GCS, she was electively intubated. The neurological examination showed neck stiffness and positive Kernig's and Brudzinski's signs. There was no papilledema, and the remainder of the examination findings were normal. The laboratory results showed a hemoglobin level of 12.5 g/dL, a total leukocyte count of 10,130 cells/µL, a platelet count of 148,000/µL, an erythrocyte sedimentation rate (ESR) of 59 mm/hour, and a C-reactive protein (CRP) level of 57.9 mg/L. Renal and liver function tests, as well as electrolyte levels, were normal. A chest X-ray revealed no significant abnormalities; the ECG displayed normal sinus rhythm. Serological tests for hepatitis B, hepatitis C, HIV, and the sputum GeneXpert were all negative. The cerebrospinal fluid (CSF) analysis showed neutrophilic pleocytosis with a total cell count of 11,342 cells/µL, a protein level of 124 mg/dL, and a glucose level of 32 mg/dL. Gram stain revealed Gram-positive cocci, and the CSF culture identified *Streptococcus pneumoniae*. CSF testing was negative for HSV-1 and -2, IgM, adenosine deaminase (ADA), and GeneXpert. Overall, the clinical presentation and CSF findings strongly supported a diagnosis of bacterial meningitis caused by *Streptococcus pneumoniae *(Table 1).

**Table 1:** Cerebrospinal fluid test results and interpretation AFB: acid-fast bacilli; CBNAAT: cartilage-based nucleic acid amplification test; ADA: adenosine deaminase; HSV1: Herpes simplex virus 1; HSV2: Herpes simplex virus 2; TB: tuberculosis

Test	Result	Reference Range	Interpretation
Total cell count	11,342 cells/µL	0-5 cells/µL	Marked pleocytosis: suggestive of severe infection or inflammation
Differential count	Neutrophil: 92%, lymphocyte: 6%	Lymphocytes 30%-70%, monocytes 15%-30%, neutrophils 0%-6%	Neutrophil predominant: highly suggestive of bacterial infection
Protein	124 mg/dL	15-45 mg/dL	Moderately elevated: suggestive of bacterial infection
Glucose	32 mg/dL	40-70 mg/dL	Decreased: suggestive of bacterial infection
Gram stain	Gram-positive cocci	Negative for organisms	Suggestive of Gram-positive bacterial infection
Culture	Streptococcus pneumoniae	No growth	Confirms bacterial growth
AFB	Negative	Negative	No evidence of tuberculosis
CBNAAT	Negative	Negative	No evidence of tuberculosis (confirming CSF-AFB results)
ADA	2.4 U/L	0- 2.5 U/L	Not suggestive of tuberculosis (TB meningitis usually have ADA levels >10 U/L)
HSV-1/HSV-2	Negative	Negative	No evidence of Herpes simplex infection
Malignant cells	Negative	Negative	No evidence of malignant cells

Diagnostic assessment and follow-up

**Figure 1:**
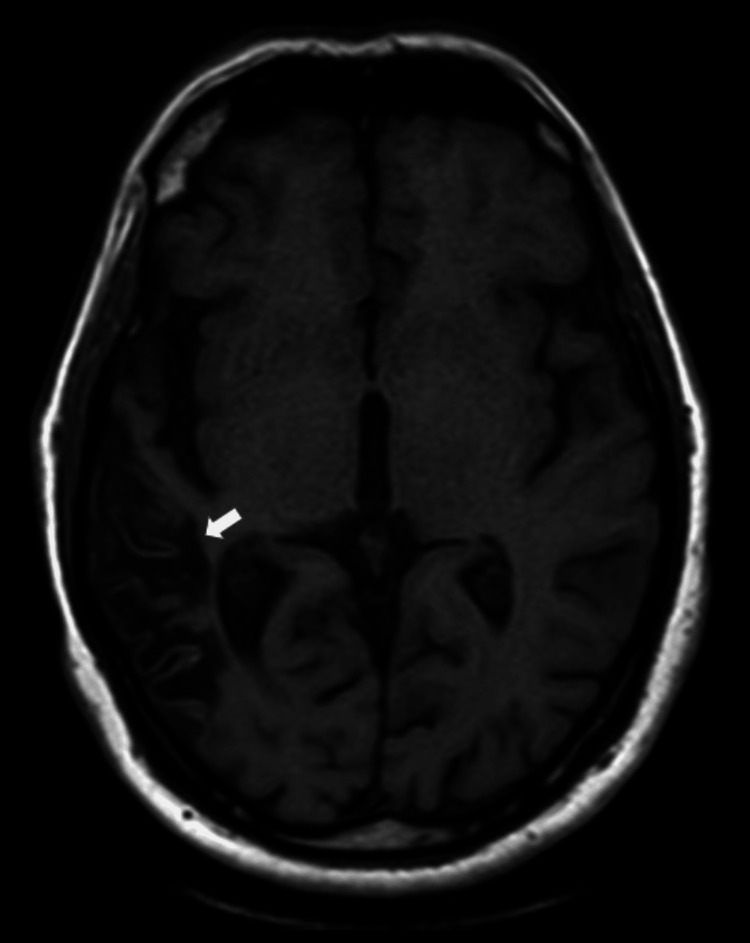
Magnetic resonance imaging (MRI) of the brain T1-weighted image (axial section) showing a hyperintense cortical lesion in the right temporal lobe

**Figure 2:**
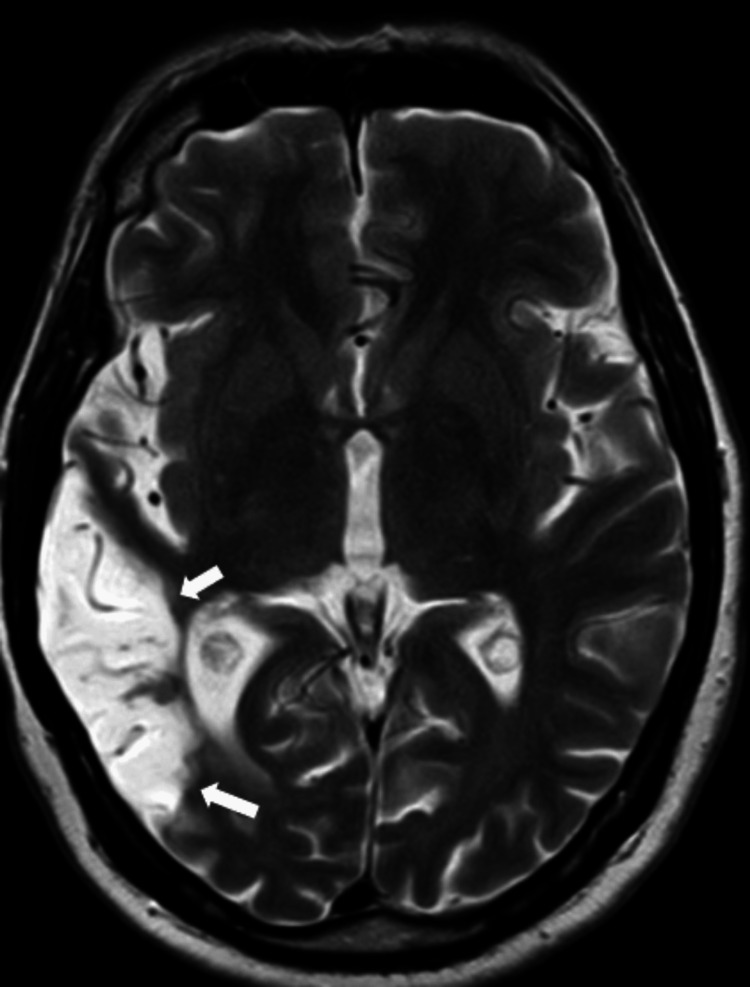
Magnetic resonance imaging (MRI) of the brain T2-weighted image (axial section) showing a hyperintense cortical lesion in the right temporal lobe

**Figure 3:**
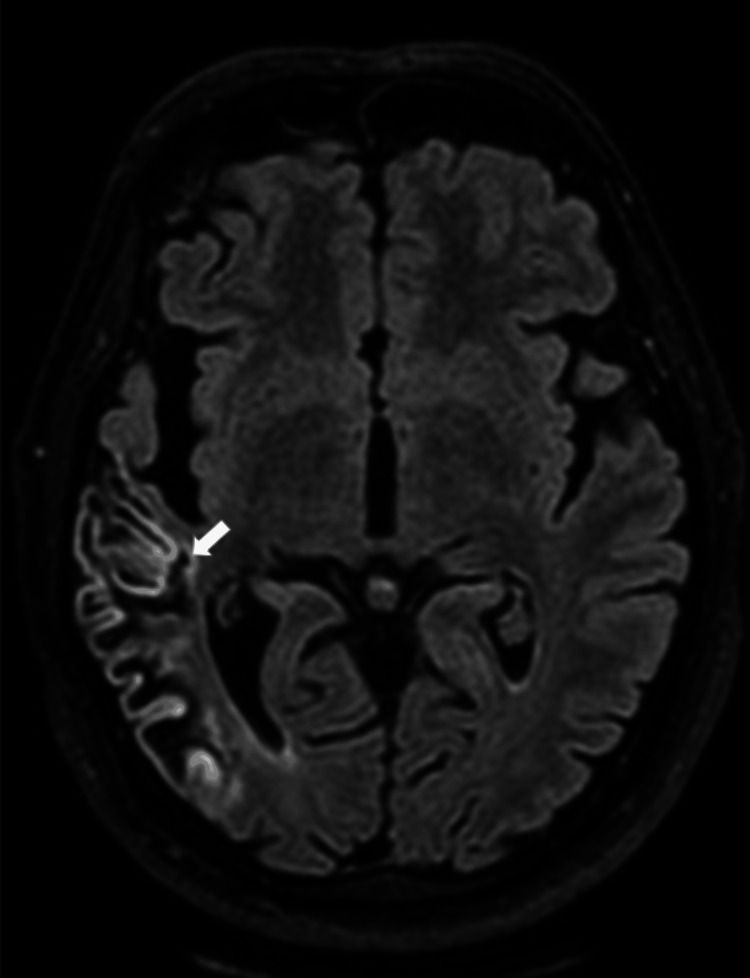
MRI brain FLAIR image (axial section) showing a hyperintense cortical lesion in the right temporal lobe FLAIR: fluid-attenuated inversion recovery; MRI: magnetic resonance imaging

Following the initiation of empirical intravenous antibiotics on day one (ceftriaxone 4 g per day and intravenous vancomycin 2 g per day for five days), antivirals (intravenous acyclovir 1.5 g per day for five days), and intravenous dexamethasone (initially 16 mg/day and tapered over two weeks), the patient's condition improved, and she was successfully weaned off the ventilator by the second day of admission. However, on the fourth day, she began experiencing worsening headaches and persistent dizziness. A repeat neurological examination revealed no focal neurological deficits, cranial nerve palsies, or abnormal pupillary response. Magnetic resonance imaging (MRI) of the brain performed on day four showed increased signal intensity on T1-weighted images (Figure 1), T2-weighted images (Figure 2), and fluid-attenuated inversion recovery (FLAIR) sequences (Figure 3) in the right temporal lobe, confirming the diagnosis of CLN. Intracranial MR venography was normal and did not show dural sinus thrombosis. Electroencephalography (EEG) was also normal, displaying non-epileptiform waves. On the sixth day, the CSF culture and sensitivity report indicated that the pathogen was resistant to third-generation cephalosporin, non-sensitive to vancomycin, and sensitive only to moxifloxacin. This prompted an appropriate change in the intravenous antibiotic treatment. Ceftriaxone and vancomycin were stopped, and the patient was administered an injection of moxifloxacin 400 mg per day for nine days.

By the ninth day of admission, the patient demonstrated significant symptomatic improvement. A repeat neurological examination revealed a GCS score of 14/15, reduced headache, and a resolution in fever. The patient was discharged at the end of the second week, with advice to continue oral moxifloxacin 400 mg per day and oral steroids for five days, and with a follow-up MRI of the brain scheduled for one month later. This case highlights the critical need to recognize CLN as a rare but severe complication of bacterial meningitis. Early detection and thorough management, including antibiotics, antivirals, and steroids, are essential for enhancing patient outcomes. Although rare, CLN should be considered in patients with bacterial meningitis who present with new or worsening neurological symptoms.

## Discussion

CLN is notably observed in cerebral infarcts, with the third layer of the cortex being particularly vulnerable to damage from moderate ischemic events. MRI brain reveals these changes, with T1-weighted images showing cortical hyperintensity, a characteristic CLN pattern. The intensity changes are also seen in FLAIR and proton density MR images [4]. During acute infarction, tissue swelling manifests as a hypointense signal on T1-weighted images. Approximately two weeks later, a bright signal begins to emerge, becoming more pronounced between one and two months before gradually diminishing by three months. In some instances, this bright signal can persist for up to 11 months [5]. Additionally, the breakdown of the blood-brain barrier caused by necrosis of the blood vessels leads to the characteristic curvilinear "gyriform" enhancement observed on gadolinium-enhanced MRI scans in cases of cortical infarction. The hyperintense signals seen on T1-weighted images can indicate the presence of substances such as hemoglobin, fat, melanin, paramagnetic substances, or protein-rich fluid. In the context of CLN, T1 hyperintensity is not attributed to hemorrhagic transformation of cerebral ischemia. Instead, it is believed to result from selective neuronal damage [6], leading to increased concentrations of proteins and other macromolecules, glial cell proliferation, and the accumulation of fat-laden macrophages in the affected cortical area. These changes enhance relaxivity by restricting the movement of water molecules, thereby causing T1 shortening.

Cerebral infarction occurs in 9%-36% of the individuals with meningoencephalitis [7]. Commonly involved arteries include the lateral and medial lenticulostriate arteries and perforators from the posterior cerebral artery, potentially impacting major arteries of the Circle of Willis. Proposed mechanisms for these infarcts include vasculitis and vasospasm, which are often observed in the middle cerebral artery territory [8]. In our case, the radiological findings can be attributed to vasculitis involving the terminal cortical branches of the right middle cerebral artery. Nonetheless, unconventional manifestations are frequently seen in central nervous system infections. The presence of leptomeningeal and parenchymal enhancement on brain MRI suggests an infectious etiology for the CLN; however, this finding was not typical in our patient. The lesions of CLN can cause persistent neurological deficits in some cases, even after the underlying cause is treated. If the cause of CLN is reversible, such as in the case of an infection, early diagnosis and timely intervention can help minimize the risk of complications.

## Conclusions

This case underscores the critical importance of recognizing CLN as a rare but serious complication of bacterial meningitis. Despite widespread immunization and antimicrobial treatment, bacterial meningitis continues to be a life-threatening condition due to such severe complications. In this instance, *Streptococcus pneumoniae* was identified as the causative agent. The characteristic delayed selective neuronal necrosis pattern highlights the utility of MRI for diagnosis and guiding prompt medical intervention. Early recognition and aggressive management are essential for mitigating severe complications such as CLN. Timely and effective treatment can lead to significant clinical improvements, as seen in the patient's gradual recovery and stabilization in the intensive care unit. The patient's progress demonstrates the effectiveness of prompt and comprehensive treatment strategies. The goal is to prevent long-term neurological damage and enhance patient outcomes.
